# Characterization of ancestral myosin XI from *Marchantia polymorpha* by heterologous expression in *Arabidopsis thaliana*

**DOI:** 10.1111/tpj.14937

**Published:** 2020-08-06

**Authors:** Zhongrui Duan, Misato Tanaka, Takehiko Kanazawa, Takeshi Haraguchi, Akiko Takyu, Atsuko Era, Takashi Ueda, Kohji Ito, Motoki Tominaga

**Affiliations:** ^1^ Faculty of Education and Integrated Arts and Sciences Waseda University 2‐2 Wakamatsu‐cho, Shinjuku‐ku Tokyo 162‐8480 Japan; ^2^ Graduate School of Science and Engineering Waseda University 2‐2 Wakamatsu‐cho, Shinjuku‐ku Tokyo 162‐8480 Japan; ^3^ Division of Cellular Dynamics National Institute for Basic Biology Nishigonaka 38, Myodaiji Okazaki Aichi 444‐8585 Japan; ^4^ Department of Basic Biology SOKENDAI Nishigonaka 38, Myodaiji Okazaki Aichi 444‐8585 Japan; ^5^ Department of Biology Graduate School of Science Chiba University Inage‐ku Chiba 263‐8522 Japan; ^6^ Department of Biological Sciences Graduate School of Science The University of Tokyo Bunkyo‐ku Tokyo 113‐0033 Japan

**Keywords:** cytoplasmic streaming, *Marchantia polymorpha*, myosin XI, heterologous expression

## Abstract

Previous studies have revealed duplications and diversification of myosin XI genes between angiosperms and bryophytes; however, the functional differentiation and conservation of myosin XI between them remain unclear. Here, we identified a single myosin XI gene from the liverwort *Marchantia polymorpha* (Mp). The molecular properties of Mp myosin XI are similar to those of Arabidopsis myosin XIs responsible for cytoplasmic streaming, suggesting that the motor function of myosin XI is able to generate cytoplasmic streaming. In cultured Arabidopsis cells, transiently expressed green fluorescent protein (GFP)‐fused Mp myosin XI was observed as some intracellular structures moving along the F‐actin. These intracellular structures were co‐localized with motile endoplasmic reticulum (ER) strands, suggesting that Mp myosin XI binds to the ER and generates intracellular transport in Arabidopsis cells. The tail domain of Mp myosin XI was co‐localized with that of Arabidopsis myosin XI‐2 and XI‐K, suggesting that all these myosin XIs bind to common cargoes. Furthermore, expression of GFP‐fused Mp myosin XI rescued the defects of growth, cytoplasmic streaming and actin organization in Arabidopsis multiple myosin XI knockout mutants. The heterologous expression experiments demonstrated the cellular and physiological competence of Mp myosin XI in Arabidopsis. However, the average velocity of organelle transport in *Marchantia* rhizoids was 0.04 ± 0.01 μm s^−1^, which is approximately one‐hundredth of that in Arabidopsis cells. Taken together, our results suggest that the molecular properties of myosin XI are conserved, but myosin XI‐driven intracellular transport *in vivo* would be differentiated from bryophytes to angiosperms.

## Significance Statement

The molecular properties and heterologous expression of* Marchantia* *polymorpha* myosin XI in Arabidopsis suggest that myosin XI is an evolutionarily conserved motor protein capable of generating cytoplasmic streaming.

## Introduction

In plants, myosin motor proteins interact with the actin cytoskeleton, playing a fundamental role in various cellular processes such as organelle movement, cytoplasmic streaming, cell division and tip growth (Boevink *et al*., 1998; Shimmen and Yokota, 2004; Abu‐Abied *et al*., 2018; Duan and Tominaga, 2018). Previous phylogenetic analyses of myosin motor domains have identified at least 79 classes of myosin in eukaryotes (Kollmar and Muhlhausen, 2017), although only two (VIII and XI) are present in plants (Odronitz and Kollmar, 2007; Bloemink and Geeves, 2011). Myosin VIII is thought to play a role in endocytosis or intercellular transport via plasmodesmata, according to its subcellular localization (Reichelt *et al*., 1999; Golomb *et al*., 2008; Haraguchi *et al*., 2014). In contrast, myosin XI is a motor protein involved in organelle transport Peremyslov *et al*., 2008; Prokhnevsky *et al*., 2008; Peremyslov *et al*., 2010; Ueda *et al*., 2010).

Phylogenetic analyses using motor domain sequences have revealed gene expansion of myosin XI from chlorophytes to angiosperms (Muhlhausen and Kollmar, 2013; Nishiyama *et al*., 2018; Haraguchi *et al*., 2019), suggesting that the functions of myosin XI have diversified throughout plant evolution. *Arabidopsis thaliana* (At), one of the model plants for angiosperms, has 13 myosin XI isoforms (XI‐1, XI‐2, XI‐A, XI‐B, XI‐C, XI‐D, XI‐E, XI‐F, XI‐G, XI‐H, XI‐I, XI‐J and XI‐K) (Reddy and Day, 2001; Tominaga and Nakano, 2012). These At myosin XIs have a similar domain composition: a motor domain with ATPase and actin‐binding activities; a neck domain to bind the myosin light chains; a coiled‐coil domain for dimerization; and a globular tail domain that binds to cargoes via adaptor proteins (Tominaga and Nakano, 2012; Tominaga and Ito, 2015). Using the motor domain, myosin XI converts the chemical energy liberated by ATP hydrolysis into physical movement along the actin filaments for organelle delivery (Tominaga *et al*., 2003; Ito *et al*., 2007, 2009).

Over the past decade, gene knockout studies have revealed that several At myosin XIs (XI‐1, XI‐2, XI‐B, XI‐I and XI‐K) are responsible for organelle movement, such as the endoplasmic reticulum (ER), Golgi stacks, peroxisomes and mitochondria (Prokhnevsky *et al*., 2008; Peremyslov *et al*., 2010; Ueda *et al*., 2010). In particular, At myosin XI‐2 and XI‐K are major motor proteins that provide the motive force for cytoplasmic streaming, active cytoplasmic flow occurring in a broad range of plant cells (Prokhnevsky *et al*., 2008). Multiple myosin XI knockouts (*xi‐1*, *xi‐2*, *xi‐b*, *xi‐i* and *xi‐k*) display defects in plant growth, suggesting that cytoplasmic streaming contributes to plant growth and development (Prokhnevsky *et al*., 2008; Peremyslov *et al*., 2010; Ojangu *et al*., 2012).

Recent studies have also shown unique functions of At myosin XI in Arabidopsis. Among the At myosin XI isoforms, XI‐I is predominantly involved in maintaining nuclear shape and movement (Tamura *et al*., 2013), while XI‐F and XI‐K play an important role in organ straightening (Okamoto *et al*., 2015) and XI‐C and XI‐E are required for organelle motility, F‐actin organization and tip growth in pollen tubes (Madison *et al*., 2015). Recently, the functional diversity of At myosin XIs was further confirmed by analyzing the tissue‐specific expression patterns, as well as the *in vitro* velocity and enzymatic activity of all 13 At myosin XI isoforms (Haraguchi *et al*., 2018). Taken together, the diversification of At myosin XI isoforms during plant evolution has led to the development of specific physiological and molecular functions.

Although the myosin XI gene family has been conserved during plant evolution, its conserved fundamental cellular functions are yet to be elucidated. For instance, it is particularly difficult to identify the conserved fundamental cellular functions of myosin XI due to functional overlap and diversity among myosin XIs in higher plants. The liverwort *Marchantia polymorpha* (Mp) is an early diverging land plant. The *Marchantia* genome has been recently sequenced, indicating low levels of genetic redundancy (Bowman *et al*., 2017; Montgomery *et al*., 2020). A detailed analysis of the phylogeny of Mp myosin XI will be valuable to identify its conserved and specialized functions throughout plant evolution.

## Results

### *Marchantia polymorpha* has a single myosin XI gene

**Figure 1:**
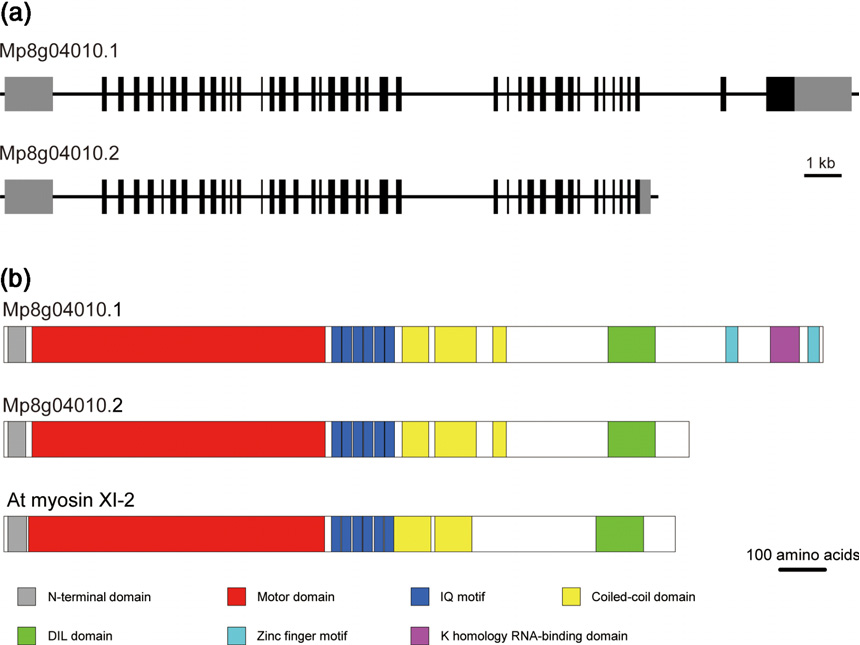
Schematic diagram of the myosin XI gene from *Marchantia polymorpha*. (a) Genomic structure of *Marchantia* myosin XI. Black boxes represent exons, black lines represent introns and gray boxes represent the untranslated regions. (b) Primary structure of *Marchantia* myosin XI with conserved protein domains (IQ, isoleucine‐glutamine).

To identify the myosin XI genes in *Marchantia* we searched the *Marchantia* genome database MarpolBase (http://marchantia.info; Bowman *et al*., 2017; Montgomery *et al*., 2020) for genes encoding for myosin XI proteins. We found a single myosin XI gene at the Mp8g04010 locus, which is predicted to yield two transcripts: Mp8g04010.1 and Mp8g04010.2 (Figure 1a). We obtained the amplified cDNA fragments as the predicted transcripts of Mp myosin XI. After sequencing, the results indicated that the cDNA of Mp8g04010.1 contained 41 exons consisting of 5511 bp. Therefore, Mp8g04010.1 was regarded to encode a 1836‐amino‐acid protein. By contrast, Mp8g04010.2 contained only 39 exons consisting of 4611 bp, as the 39th exon contained a termination codon. Therefore, Mp8g04010.2 was considered to encode a 1536‐amino‐acid protein. Both the predicted translation products have an N‐terminal domain, a motor domain (ATPase), six isoleucine–glutamine (IQ) motifs, coiled‐coil domains and a DIL domain (Figure 1b). In addition to these domains, the protein encoded by Mp8g04010.1 also has two Zn‐finger motifs and a K‐homology RNA‐binding domain at its carboxyl‐terminal region (Figure 1b). Because the short C‐terminal structure of Mp8g04010.2 was common to Mp myosin XI and At myosin XI‐2 (Figure 1b), this short variant was deemed suitable to investigate the common functions between Mp myosin XI and At myosin XI.

### Velocity and actin‐activated ATPase activities of Mp myosin XI

**Figure 2:**
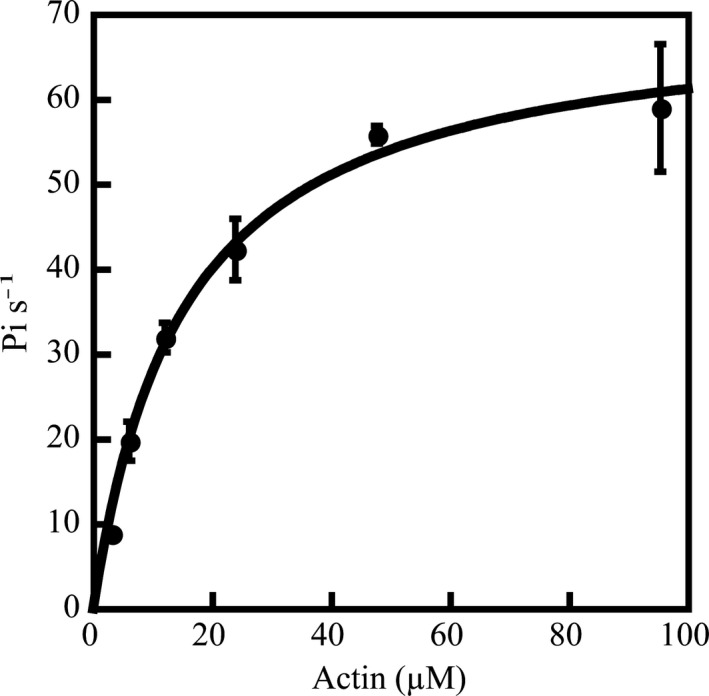
Actin‐activated Mg^2+^‐ATPase activity of *Marchantia polymorpha* (Mp) myosin XI‐MD. The actin‐activated Mg^2+^‐ATPase activities of Mp myosin XI‐MD were measured in the presence of various F‐actin concentrations. The mean ± SEM data fitted closely to the Michaelis–Menten equation. *V*_max_ and *K*_m_ values were 71 ± 2.9 Pi s^−1^ and 15 ± 1.9 μm, respectively. Values are averages of three assays using two independent preparations of Mp myosin XI‐MD.

Figure S1 in the online Supporting Information shows a schematic diagram of full‐length Mp myosin XI (Mp8g04010.2), deduced from its amino acid sequence. To measure the velocity of Mp myosin XI, we generated a recombinant full‐length myosin XI construct (Mp myosin XI‐Full; Figure S1). This construct was co‐expressed with Arabidopsis calmodulin in insect cells, then purified using nickel‐ and FLAG‐affinity resins (Ito *et al*., 2007, 2009; Haraguchi *et al*., 2016, 2018). The actin‐sliding velocity of Mp myosin XI‐Full measured using an *in vitro* actin‐gliding assay was 4.2 ± 0.43 µm sec^−1^ (mean ± SEM). This was similar to the value for At myosin XI‐2, a motor protein providing the motive force for cytoplasmic streaming in Arabidopsis (Umeki *et al*., 2009; Trybus, 2012; Tominaga *et al*., 2013; Haraguchi *et al*., 2018). Several previous studies on non‐plant myosins reported that actin‐activated ATPase activities are often inhibited by the free tail domain of full‐length myosin due to a folded conformation (Stoffler and Bahler, 1998; Krementsov *et al*., 2004; Umeki *et al*., 2009). To avoid the inhibited state of full‐length myosin XI in the absence of cargo, actin‐activated ATPase activity was measured using a recombinant motor domain of Mp myosin XI (Mp myosin XI‐MD). The plot of ATPase activity against actin concentration closely fitted a Michaelis–Menten‐type curve (Figure 2). From the curve, we determined the maximum rate of ATP turnover (*V*_max_) and the actin concentration at which the ATPase rate reached 50% of the maximum rate (*K*_m_), which were 71 ± 2.9 Pi s^−1^ and 15 ± 1.9 μm, respectively; and similar to the values for At myosin XI‐2 (Haraguchi *et al*., 2018).

### *Marchantia* myosin XI localizes to motile subcellular structures along actin filaments in cultured Arabidopsis cells

**Figure 3:**
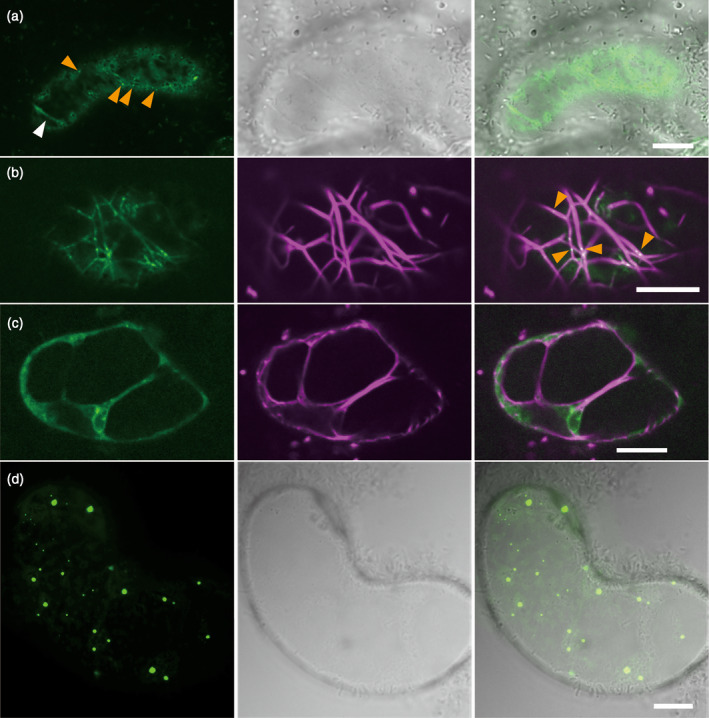
Transient expression of *Marchantia polymorpha* (Mp) myosin XI in Arabidopsis cultured cells. (a) GFP‐fused full‐length Mp myosin XI (GFP‐Mp myosin XI‐Full) expression in the Arabidopsis cultured cell line Alex. The punctate structures of GFP‐Mp myosin XI‐Full are shown by orange arrowheads and the strand‐like structures by white arrowheads. (b) Co‐expression of GFP‐Mp myosin XI‐Full (green) and Lifeact‐TagRFP (magenta) in the cortical region. The co‐localization of the punctate structures and F‐actin is shown by orange arrowheads. (c) Co‐expression of GFP‐Mp myosin XI‐Full (green) and Lifeact‐TagRFP (magenta) in the central region. The co‐localization of the strand‐like structures and F‐actin is shown by a white arrowhead. (d) GFP‐fused Mp myosin XI‐tail domain (GFP‐Mp myosin XI‐tail) was transiently expressed in Arabidopsis Alex cells. Scale bars = 10 μm.

We constructed a plasmid that contained GFP‐fused full‐length Mp myosin XI cDNA of Mp8g04010.2 (hereafter, GFP‐Mp myosin XI‐Full; Figure S1). This gene was then transiently expressed, driven by the cauliflower mosaic virus (CaMV) 35S promoter in the Arabidopsis cultured cell line Alex. GFP‐Mp myosin XI‐Full was observed as punctate and strand‐like structures moving along the linear tracks, while immotile diffused GFP signals were observed in the cytosol (Figure 3a, Movie S1). To determine whether the motile subcellular localizations effectively reflected the distribution of Mp myosin XIs along the actin filaments, TagRFP‐Lifeact, a probe visualizing filamentous actin (F‐actin) (Era *et al*., 2009), was co‐expressed with GFP‐Mp myosin XI‐Full in Arabidopsis Alex cells. The punctate and the strand‐like structures of GFP‐Mp myosin XI‐Full were partially co‐localized with TagRFP‐Lifeact (Figure 3b and 3c), suggesting that Mp myosin XI‐binding structures associate with Arabidopsis F‐actin. We then treated the GFP‐Mp myosin XI‐Full‐expressing Arabidopsis Alex cells with 20 μm latrunculin B, an inhibitor of actin polymerization. The GFP‐Mp myosin XI‐Full did not form strand‐like structures but exhibited many immotile punctate structures of various sizes following the addition of latrunculin B for 10 min (Figure S2a). When cells were treated for 60 min, GFP‐Mp myosin XI‐Full formed large puncta instead of small punctate structures (Figure S2b), suggesting that these smaller structures congregate during long‐term treatment. This pharmacological experiment supports the hypothesized interaction between Mp myosin XI and F‐actin. Because the motor domain of myosin interacts with actin, we used the tail domain, a motorless form, to verify the interaction between the motor domain of Mp myosin XI and actin. We transiently expressed the GFP‐fused Mp myosin XI‐tail domain of Mp8g04010.2 (hereafter, GFP‐Mp myosin XI‐tail) driven by the CaMV 35S promoter in Arabidopsis Alex cells (Figure S1). The GFP‐Mp myosin XI‐tail formed immotile punctate structures that were similar to those of GFP‐Mp myosin XI‐Full when treated with latrunculin B (Figure 3d), suggesting that motorless Mp myosin XI acts as a dominant negative. Moreover, the strand‐like structures observed in both experiments were not detected in the GFP‐Mp myosin XI‐tail‐expressing cells. These results indicate that the interaction between the motor domain of Mp myosin XI and F‐actin could not only contribute to the transport of Mp myosin XI‐binding cargoes but may also sustain the strand‐like cargo structure.

### Co‐localization of Mp myosin XI with the ER

**Figure 4:**
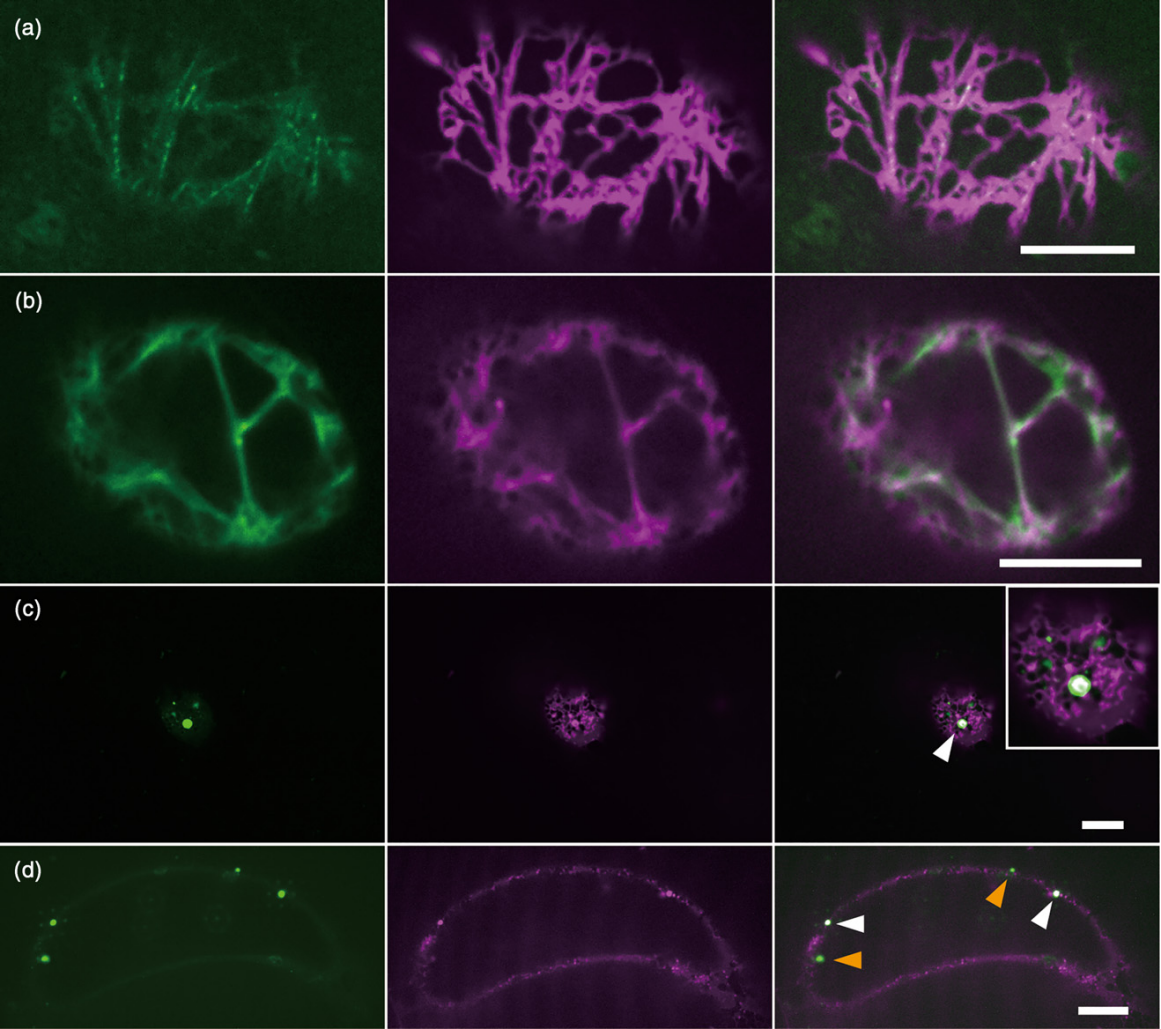
Co‐expression of GFP‐Mp myosin XI and the TagRFP endoplasmic retuculum (ER) marker. GFP‐Mp myosin XI‐Full and GFP‐Mp myosin XI‐tail (green) co‐expression with the TagRFP ER marker (SP‐TagRFP‐HDEL, magenta) in Arabidopsis Alex cells. Co‐localization of: (a) GFP‐Mp myosin XI‐Full punctate structures with TagRFP ER strands in the cortical region; (b) GFP‐Mp myosin XI‐Full strand‐like structures with transvacuolar ER strands in the central region; (c) GFP‐Mp myosin XI‐tail and aggregated ER structures in the cortical region (white arrowheads; the white box shows a magnified image); and (d) GFP‐Mp myosin XI‐tail and aggregated ER structures in the central region (other GFP‐Mp myosin XI‐tails were not co‐localized with ER, orange arrowheads). Scale bars = 10 μm.

In Arabidopsis cells, the ER develops a static peripheral network and rapidly moving strand‐like structures. Gene knockout analyses of Arabidopsis previously revealed that the motility of myosin XIs is involved in the dynamics and morphology of the ER (Ueda *et al*., 2010). GFP‐Mp myosin XI‐Full and an ER‐targeting marker, SP‐TagRFP‐HDEL (Mitsuhashi *et al*., 2000), were transiently co‐expressed in Arabidopsis Alex cells to verify their interaction. In the cortical region of the cells, the motile punctate structures of GFP‐Mp myosin XI‐Full along the filamentous tracks were co‐localized with moving ER strands but did not overlap with the reticulated ER sheets (Figure 4a, Movie S2). In the central region of the cells, the strand‐like structures of GFP‐Mp myosin XI‐Full were co‐localized with transvacuolar ER strands (Figure 4b). These results suggest that ER is a possible cargo for Mp myosin XI. Because the tail domain of myosin mediates cargo attachment (i.e. organelles), overexpression of the myosin tail domain is a useful tool for identifying the organelles to which it binds as it acts in a dominant negative fashion and inhibits the actin‐dependent movement of organelles. In our experiment, when the ER marker was co‐expressed with GFP‐Mp myosin XI‐tail, aggregated ER structures emerged in the reticulated ER structure in the cortical region, while moving ER strands disappeared. The aggregated ER structures also overlapped with the GFP‐Mp myosin XI‐tail (Figure 4c), suggesting that Mp myosin XI is involved in movement of the ER and its structural maintenance. Furthermore, the transvacuolar ER strands disappeared with the expression of GFP‐Mp myosin XI‐tail. Aggregated ER structures were also observed in the central region, co‐localized with Mp myosin XI‐tail (Figure 4d, white arrowheads). These results suggest either direct or indirect physical associations between the ER and Mp myosin XI. However, some GFP‐Mp myosin XI‐tails did not co‐localize with the ER, suggesting interactions between Mp myosin XI and structure(s) other than the ER (Figure 4d, orange arrowheads).

It has also been reported that the movement of Golgi bodies and peroxisomes is dependent on myosin XI (Peremyslov *et al*., 2010). To examine the interactions between Mp myosin XI and other membranous organelles, a Golgi body‐targeting marker, ST‐mRFP (Boevink *et al*., 1998), and a peroxisome‐targeting marker, TagRFP‐H1 (Mano *et al*., 1999), were individually co‐expressed with GFP‐myosin XI. Neither co‐localized with GFP‐Mp myosin XI‐Full nor with GFP‐Mp myosin XI‐tail (Figure S3).

### Co‐localization of Mp myosin XI with At myosin XI and cytoplasmic streaming

**Figure 5:**
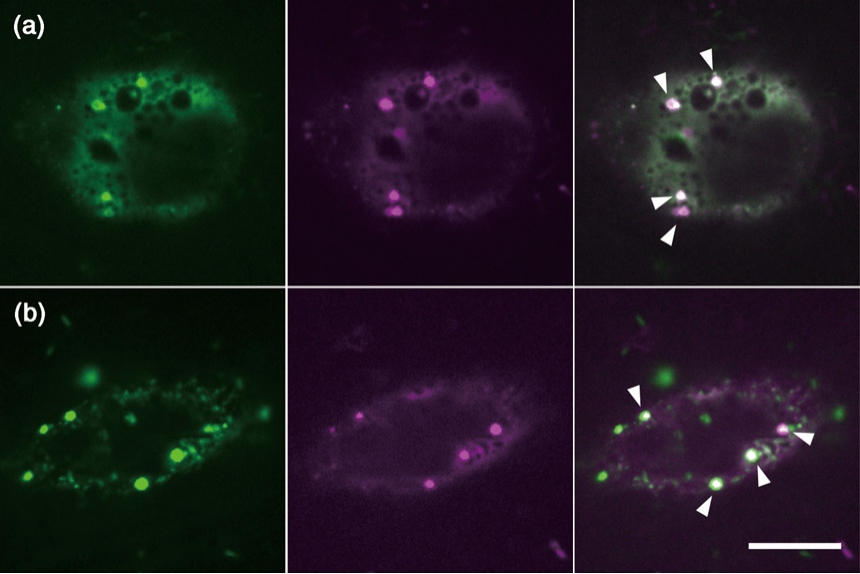
Co‐expression of *Marchantia polymorpha* (Mp) myosin XI‐tail and the tail domain of At myosin XIs. (a) The GFP‐fused tail domain of *Arabidopsis thaliana* (At) myosin XI‐K (green) and the TagRFP‐fused tail domain of Mp myosin XI (magenta) expression in Arabidopsis Alex cells. Co‐localization of large puncta of At myosin XI‐K‐tail and Mp myosin XI‐tail (white arrowheads). (b) The GFP‐fused tail domain of At myosin XI‐2 (green) and the TagRFP‐fused tail domain of Mp myosin XI (magenta) expression in Arabidopsis Alex cells. Co‐localization of large puncta of the At myosin XI‐2‐tail and Mp myosin XI‐tail (white arrowheads). Scale bar = 10 μm.

To determine whether At myosin XIs and Mp myosin XI interact with the same organelles, TagRFP‐Mp myosin XI‐tail was co‐expressed with GFP‐fused At myosin XI‐tails of several At myosin XI members in Arabidopsis Alex cells. Given the possibility of Mp myosin XI acting as a motive force for cytoplasmic streaming, we selected At myosin XI‐K and XI‐2 – the major motive forces of cytoplasmic streaming – from the 13 At myosin XI members. Both At myosin XI‐K and At myosin XI‐2 tails localized to punctate structures with diffused localization in the cytosol (Figure 5a,b) as reported in Avisar *et al*. (2009). Strikingly, Mp myosin XI‐tail localized to the same large puncta as both At myosin XI‐K and XI‐2 (Figure 5a,b, white arrowheads). These results suggested that Mp myosin XI may bind to the same cargo(es) as At myosin XI‐K and XI‐2, which could act as a motive force for cytoplasmic streaming (Peremyslov *et al*., 2008).

### Cytoplasmic streaming generated by Mp myosin XI contributes to the growth of Arabidopsis

**Figure 6:**
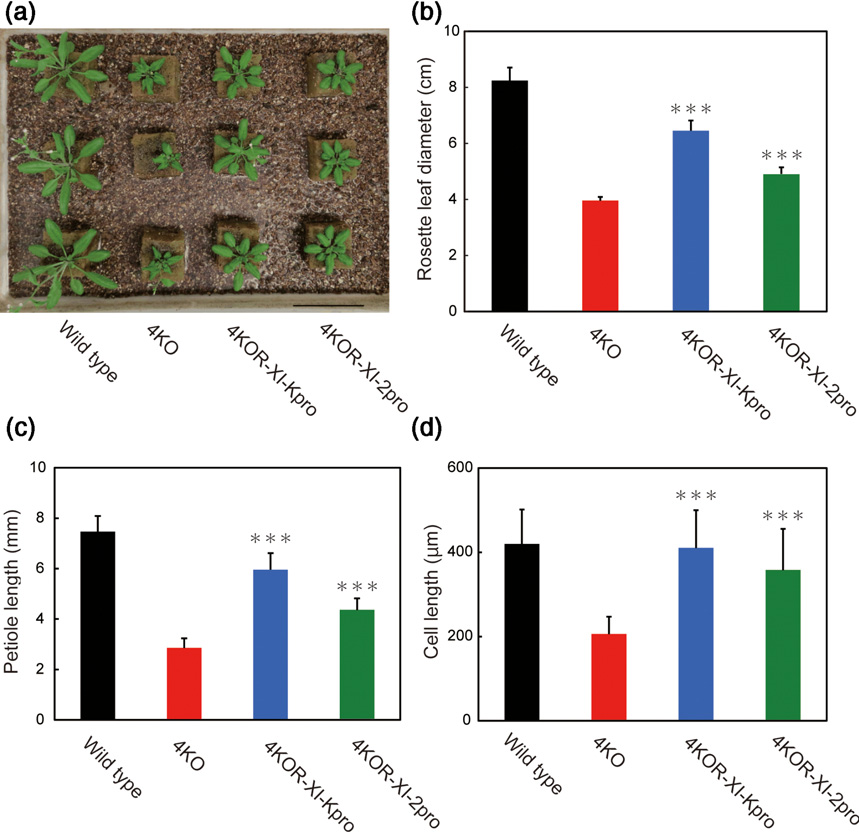
Phenotypic analyses of quadruple *Arabidopsis thaliana* (At) myosin XI knockout (4KO) mutant plants expressing GFP‐Mp myosin XI‐Full driven by At myosin XI‐2 or At XI‐K promoter. (a) Representative image of 28‐day‐old wild‐type *xi‐1 xi‐2 xi‐k xi‐i* knockout (4KO), 4KO expressing XI‐Kpro‐GFP‐Mp myosin XI (4KOR‐XI‐Kpro) and 4KO expressing XI‐2pro‐GFP‐Mp myosin XI (4KOR‐XI‐2pro) plants. Scale bar = 5 cm. (b) Mean rosette leaf diameters of 28‐day‐old wild‐type, 4KO, 4KOR‐XI‐Kpro and 4KOR‐XI‐2pro plants (mean ± SEM, *n* = 8). (c) Petiole length of the third leaves (mean ± SEM, *n* = 10). (d) Cell length of epidermal cells in the third leaf (mean ± SEM, *n* = 40). ^***^ *P* < 0.001 by Student's *t*‐test compared with 4KO.

Myosin XI multiple mutants (among *xi‐1*, *xi‐2*, *xi‐b*, *xi‐i* and *xi‐k*) of Arabidopsis display severe growth defects as a result of inhibited organelle trafficking, indicating that myosin XI plays a critical role in cytoplasmic streaming and is hence a key factor in Arabidopsis growth (Peremyslov *et al*., 2010). To determine whether Mp myosin XI plays a similar physiological roles in Arabidopsis plants, we examined the effect of the heterologous expression of GFP‐Mp myosin XI‐Full, driven by the At myosin XI‐2 or XI‐K promoters, in quadruple At myosin XI knockout mutant plants (4KO; *xi‐1 xi‐2 xi‐k xi‐i*). The phenotypes were confirmed in five independent transgenic Arabidopsis lines. Intriguingly, the defect of growth in the 4KO mutant was partially rescued by the expression of Mp myosin XI driven by At myosin XI‐K (4KOR‐XI‐Kpro) and the At myosin XI‐2 promoter (4KOR‐XI‐2pro) (Figure 6a). An investigation into the phenotypes of these heterologously expressed plants revealed a significant degree of restoration of the rosette leaf diameter and petiole length compared with 4KO plants (Figure 6b,c). A previous study has reported that the shorter petiole length in 4KO plants was due to a decrease in cell length in the petiole (Peremyslov *et al*., 2010). The length of epidermal cells of the third leaf was measured to investigate the effect of Mp myosin XI on cell growth. Epidermal cell length in 4KOR‐XI‐Kpro and 4KOR‐XI‐2pro plants was approximately 2.0‐fold and 1.7‐fold greater than that of 4KO, respectively (Figure 6d); this ratio was somewhat consistent with that of petiole length (Figure 6c). These results indicate that the restoration of plant size in the heterologously expressed plants was due to the corresponding increase in cell size.

**Figure 7:**
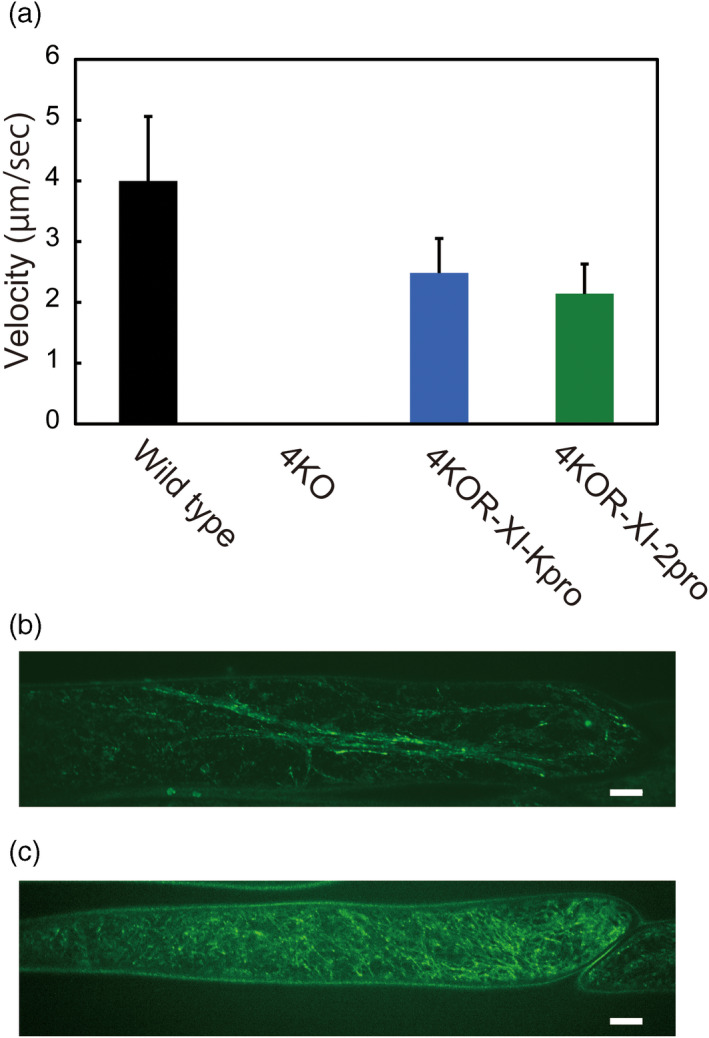
Cytoplasmic streaming in petiole epidermal cells. (a) Cytoplasmic streaming velocity in epidermal cells of the third leaf petiole. (b) Subcellular localization of GFP‐Mp myosin XI in petiole epidermal cells of 4KOR‐XI‐Kpro plants. Scale bar = 10 μm. (c) Subcellular localization of GFP‐Mp myosin XI in the petiole epidermal cells of 4KOR‐XI‐2pro plants. Scale bar = 10 μm. Values are mean ± SEM (*n* = 30).

The velocity of cytoplasmic streaming, which is dependent on the velocity of myosin XIs, is directly proportional to plant growth (Tominaga *et al*., 2013). Epidermal cells of the third leaf petiole were observed microscopically to test the effect of GFP‐Mp myosin XI‐Full on cytoplasmic streaming (Figure 7a). Continuous cytoplasmic streaming was detected in the epidermal cells of wild‐type plants at an average velocity of 4.0 ± 1.1 μm sec^−1^ (Movie S3). By contrast, the continuous flow of cytoplasm was almost undetectable in 4KO plants (Movie S4). Strikingly, continuous cytoplasmic streaming was observed in the epidermal cells of 4KOR‐XI‐Kpro and 4KOR‐XI‐2pro plants at velocities of 2.5 ± 0.6 and 2.1 ± 0.5 μm sec^−1^, respectively (Movies S5 and S6). Subcellular localization of GFP‐Mp myosin XI‐Full was also observed using confocal laser scanning microscopy in petiole epidermal cells of 4KOR‐XI‐Kpro and 4KOR‐XI‐2pro plants. GFP‐Mp myosin XI‐Full formed many punctate structures of various sizes (Figure 7b,c), similar to observations of Arabidopsis Alex cells. The difference in brightness of the signal from the two lines is due to the different expression levels of GFP‐Mp myosin XI‐Full with different activities of the XI‐K and the XI‐2 promoter. Time‐lapse imaging showed the active movement of GFP‐Mp myosin XI‐Full in both 4KOR‐XI‐Kpro (Movie S7) and 4KOR‐XI‐2pro (Movie S8) plants, at average velocities of 2.2 ± 0.5 and 1.9 ± 0.4 μm sec^−1^, respectively, which is consistent with the cytoplasmic streaming velocity in heterologously expressed plants (Movies S5 and S6).

### Mp myosin XI in Arabidopsis actin organization

**Figure 8:**
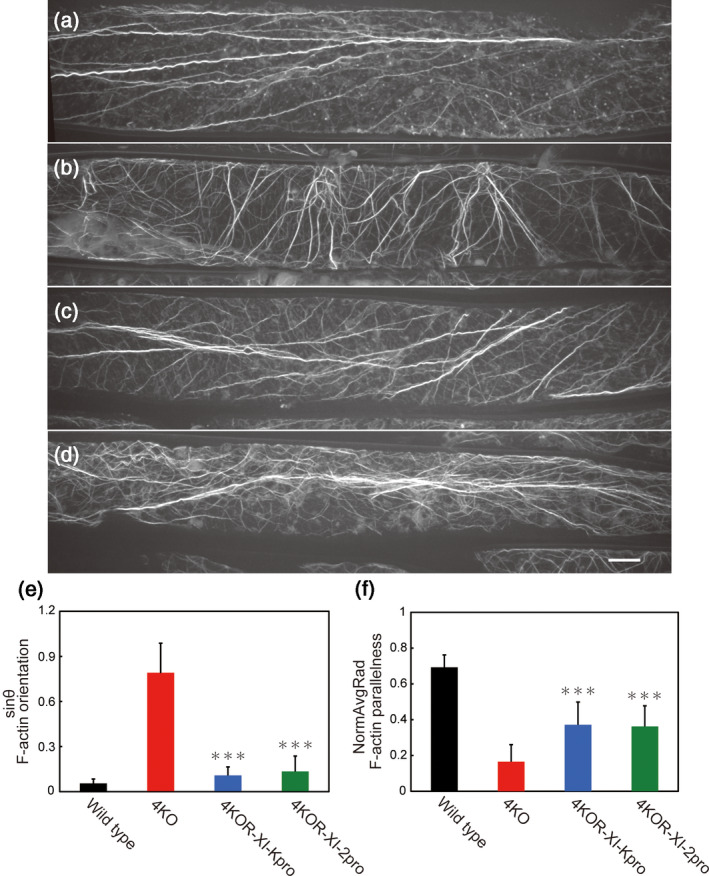
F‐actin organization in epidermal cells of the third leaf petiole. Representative confocal images of phalloidin staining for F‐actin in the petiole epidermal cells of wild‐type (a), quadruple *Arabidopsis thaliana* (At) myosin XI knockout (4KO) (b), 4KOR‐XI‐Kpro (c) and 4KOR‐XI‐2pro (d) plants. Scale bar = 10 µm. Quantitative analyses of the F‐actin architecture in epidermal cells of wild‐type (black), 4KO (red), 4KOR‐XI‐Kpro (blue) and 4KOR‐XI‐2pro (green) (mean ± SEM, *n* = 30 cells from five plants). (e) sinθ reflecting F‐actin orientation. (f) NormAvgRad, reflecting the parallelness of F‐actin. Scale bars = 10 µm. ^***^ *P* < 0.001 by Student's *t*‐test compared with 4KO.

In Arabidopsis, F‐actin is more transversely oriented in the triple and quadruple myosin XI knockouts (among *xi‐1*, *xi‐2*, *xi‐b*, *xi‐i* and *xi‐k*) compared with the typical longitudinal orientation in wild‐type cells (Peremyslov *et al*., 2010). The results indicate that these At myosin XIs are required for actin organization. To confirm whether Mp myosin XI can alter actin organization in Arabidopsis, we observed F‐actin in wild‐type, 4KO, 4KOR‐XI‐Kpro and 4KOR‐XI‐2pro plants using Alexa Fluor 488 phalloidin staining. Thick and longitudinal F‐actin bundles were observed in petiole epidermal cells of the wild type (Figure 8a). In contrast, most of the F‐actin bundles in 4KO plants were randomly oriented (Figure 8b). In 4KOR‐XI‐Kpro and 4KOR‐XI‐2pro plants, F‐actin bundles were oriented more regularly than those in 4KO plants (Figure 8c, d). The organization of F‐actin was quantified using two parameters: sinθ and NormAvgRad. Sinθ, ranging from 0 to 1, is a parameter for the orientation of the F‐actin bundles. The maximum value occurred when F‐actin bundles showed complete transverse orientation while the lowest value occurred when they showed complete longitudinal orientation. NormAvgRad, ranging from 0 to 1, is a parameter for the parallelness of F‐actin bundles. The value for this parameter was lowest when F‐actin bundles had a random orientation but highest when they ran parallel to each other. Both parameters indicated that F‐actin was oriented more randomly in 4KO than in wild‐type plants. By contrast, in 4KOR‐XI‐Kpro and 4KOR‐XI‐2pro plants, the wild‐type orientation and parallel arrangement of F‐actin bundles were partially restored by the expression of GFP‐Mp myosin XI‐Full (Figure 8e,f). These results demonstrated that Mp myosin XI can contribute to actin organization in Arabidopsis.

### Cytoplasmic streaming in *Marchantia* rhizoids

In our previous study we were unable to detect definitive cytoplasmic streaming in dorsal thalli cells in *Marchantia* (Era *et al*., 2009). In this study, a continuous flow of cytoplasm and tip growth was observed in *Marchantia* rhizoids (Movie S9). To measure the velocity of organelles in *Marchantia* rhizoids, we examined the motility of mitochondria using MitoTracker Orange staining (Movie S10). The mean velocity of mitochondria was 0.04 ± 0.01 μm sec^−1^ (*n* = 40). The results indicate that the velocity of cytoplasmic streaming in *Marchantia* rhizoids is approximately one‐hundredth of that in Arabidopsis cells.

## Discussion

Thirteen Arabidopsis myosin XI genes have been shown to exhibit functional diversity and redundancy (Duan and Tominaga, 2018; Haraguchi *et al*., 2018). In this study, our examination of the *Marchantia* genome identified a single Mp myosin XI gene, which can shed light on the conserved fundamental cellular functions of myosin XI during evolution. *In vitro* analyses of Mp myosin XI revealed the conserved molecular properties of myosin XI during land plant evolution. Previously, an *in vitro* assay using purified myosin XI from plants indicated that myosin XI velocity was consistent with that of cytoplasmic streaming in plant cells (Tominaga *et al*., 2003; Ito *et al*., 2007; Tominaga *et al*., 2013). Our *in vitro* actin‐gliding assay showed that the velocity of Mp myosin XI‐Full was consistent with that of cytoplasmic streaming in Arabidopsis (Tominaga *et al*., 2013). The velocity and actin‐activated ATPase activity of Mp myosin XI were similar to those of At myosin XIs responsible for cytoplasmic streaming (Haraguchi *et al*., 2018). These molecular properties imply that Mp myosin XI could be responsible for the generation of cytoplasmic streaming.

The transient expression of GFP‐fused full‐length Mp myosin XI (GFP‐Mp myosin XI‐Full) in cultured Arabidopsis cells revealed that Mp myosin XI binds to subcellular structures moving along the F‐actin. This active movement depends on the interaction between Mp myosin XI and Arabidopsis F‐actin. Previous studies have indicated that ER active transport mediated by myosin XIs might drive cytoplasmic streaming in Arabidopsis Ueda *et al*., 2010; Ueda *et al*., 2015). In this study, the transient expression of GFP‐Mp myosin XI‐Full demonstrated the co‐localization and simultaneous movements between Mp myosin XI‐binding cargoes and ER strands in Arabidopsis cultured cells (Figures 4 and S2). These observations suggest the cellular competency of Mp myosin XI for generating cytoplasmic streaming in Arabidopsis cells.

Among the 13 At myosin XI isoforms, At myosin XI‐2 and XI‐K are the major motor proteins providing the motive force for cytoplasmic streaming (Peremyslov *et al*., 2008). Given that Mp myosin XI is also potentially a motive force for cytoplasmic streaming, the tail domain of TagRFP‐Mp myosin XI was co‐expressed with that of GFP‐At myosin XI‐2 and XI‐K in Arabidopsis cultured cells (Figure 5). The co‐localizations of Mp myosin XI and those At myosin XIs suggest that Mp myosin XI shares the same cargoes as At myosin XI‐2 and K, mediated by specific Arabidopsis receptors such as MyoBs (Peremyslov *et al*., 2012; Stephan *et al*., 2014). The transient expression of GFP‐Mp myosin XI‐Full in cultured Arabidopsis cells showed that it was localized to a variety of subcellular structures (Figure 3a, Movie S1). Previously, expression of full‐length YFP‐At myosin XI‐K revealed that this myosin XI is associated with the motile membranous vesicle‐like compartments (Peremyslov *et al*., 2012; Stephan *et al*., 2014). The size and shape of these vesicle‐like compartments of At myosin XI‐K are similar to the smaller punctate structures that had been observed after the expression of GFP‐Mp myosin XI‐Full. By contrast, larger intracellular and strand‐like structures observed after the expression of GFP‐Mp myosin XI‐Full have not been reported in YFP‐At myosin XI‐K expressing cells. This difference in localization between Mp myosin XI and At myosin XI‐K suggests that they are not completely redundant.

In Arabidopsis, the quadruple knockout mutant 4KO (*xi‐1 xi‐2 xi‐k xi‐i*) had considerable growth defects due to the absence of continuous organelle movement (Peremyslov *et al*., 2010) and cytoplasmic streaming (Movie S4). To determine the physiological competence of Mp myosin XI in Arabidopsis, we heterologously expressed cDNA of GFP‐Mp myosin XI‐Full, driven by an At myosin XI promoter (At myosin XI‐2 or XI‐K promoters), in 4KO. Interestingly, the heterologous expression of GFP‐myosin XI‐Full induced continuous cytoplasmic streaming in 4KO epidermal cells (Figure 7a, Movies S5 and S6). In these cells, GFP‐Mp myosin XI was localized to some intracellular structures and actively moved at the velocity of cytoplasmic streaming (Figure 7b,c, Movies S7 and S8). These localizations and movements were comparable to those observed in GFP‐Mp myosin XI‐expressing Arabidopsis cultured cells. Furthermore, Mp myosin XI‐driven cytoplasmic streaming partially compensated for the defects of plant growth in 4KO (Figure 6), indicating that it can contribute to plant growth in Arabidopsis in a similar way to At myosin XIs in previous studies (Peremyslov *et al*., 2010; Tominaga *et al*., 2013).

Previous studies have demonstrated that multiple myosin XI knockout mutants in Arabidopsis caused disorganization of actin in epidermal cells (Peremyslov *et al*., 2010; Ueda *et al*., 2010). This phenomenon has been explained using a three‐way interaction model: the ER network linked to F‐actin via myosin XIs (XI‐2, XI‐K and XI‐1) may play a role in generating forces for changes in the shape of F‐actin during actin remodeling (Ueda *et al*., 2015). In this study, image analyses revealed that the random actin organization of 4KO is partially compensated for by the heterologous expression of Mp myosin XI (Figure 8). This result suggests that Mp myosin XI transports ER as a cargo and possesses redundant functions with At myosin XI‐2 and K for F‐actin organization, mediated by the myosin XI–ER association. Taken together, our results for heterologous expression of Mp myosin XI confirmed the physiological competence of Mp myosin XI in Arabidopsis.

Recently obtained genomic information from various plants has revealed that myosin XI genes diversified throughout the course of land plant evolution (Muhlhausen and Kollmar, 2013; Nishiyama *et al*., 2018). We identified a single myosin XI gene in *Marchantia*, belonging to a group of basal land plants. By contrast, angiosperms have around 10 myosin XI genes (Peremyslov *et al*., 2011; Muhlhausen and Kollmar, 2013). Compared with *Marchantia*, angiosperms have various specific plant tissues, such as vascular tissues and phytomer structures of shoots with reproductive tissues. Our recent study revealed variation in tissue‐specific expression patterns among the 13 At myosin XIs, including the vascular‐specific At myosin XI and pollen‐specific At myosin XI (Haraguchi *et al*., 2018). These findings suggest that diversification of myosin XI could be correlated with the complex developmental systems of angiosperms. Interestingly, the classification based on tissue‐specific expression patterns of At myosin XIs appears to be roughly correlated with their *in vitro* velocities (Haraguchi *et al*., 2018). The ubiquitously expressed At myosin XIs (XI‐1, −2, ‐B and ‐K), which are the motive force for cytoplasmic streaming, generate velocities of around 5–7 µm sec^−1^, while the specifically pollen‐ or vascular bundle‐expressed At myosin XIs (XI‐A, ‐C, ‐D, ‐E, ‐F and ‐G) generate higher velocities (12–23 µm sec^−1^). Only At XI‐I, responsible for nuclear movement and shape, generates a low velocity (0.1 µm sec^−1^), suggesting that the diversification of molecular functions and expression patterns of myosin XIs in angiosperms results in the achievement of their specific tasks during the process of plant evolution. In this study, the *in vitro* velocity of the single Mp myosin XI is comparable with that of cytoplasmic streaming in Arabidopsis (4 µm sec^−1^). The results for heterologous Mp myosin XI expression in Arabidopsis strongly suggest that the generation of cytoplasmic streaming is a conserved fundamental cellular function of myosin XI. Although the conserved molecular properties and competence of Mp myosin XI in Arabidopsis were confirmed, the velocity of Mp myosin XI‐driven cytoplasmic streaming in Arabidopsis and that of cytoplasmic streaming in *Marchantia* were significantly different, indicating that Mp myosin XI does not generate cytoplasmic streaming in *Marchantia* as well as in Arabidopsis. Mp myosin XI, therefore, generates rapid intracellular transport other than cytoplasmic streaming in *Marchantia* cells. This study suggests that the molecular properties of myosin XI are conserved, but that myosin XI‐driven intracellular transport would be differentiated between bryophytes and angiosperms.

In this study, slow cytoplasmic streaming (0.04 ± 0.01 μm sec^−1^) was observed in *Marchantia* rhizoids. The moss species *Physcomitrella patens* also shows slow organelle motility at a similar velocity (Furt *et al*., 2012). Despite the fact that no fast cytoplasmic streaming has been found in moss, the fact that the molecular function of myosin XI is conserved from moss to higher plants is an interesting discovery when considering the evolution of intracellular transport in plants. Previous studies revealed that *Physcomitrella* myosin XIs are essential for tip growth in protonemal cells (Vidali *et al*., 2010; Furt *et al*., 2013). Recently, Mp myosin XI mutants were isolated as shorter‐rhizoid mutants in a large‐scale forward genetic screening (Honkanen *et al*., 2016), indicating that Mp myosin XI contributes to tip growth of *Marchantia* rhizoids. Therefore, myosin XI would make a major contribution to tip growth but not to rapid cytoplasmic streaming in bryophyte cells. Previous studies suggest the delivery of organelles and vesicles by myosin XI is required for tip growth (Hapler *et al*., 2001; Vidali *et al*., 2010; Cole and Fowler, 2006; Ojangu *et al*., 2007; Prokhnevsky *et al*., 2008; Cai and Cresti, 2009; Sparkes, 2010; Furt *et al*., 2013). To identify the cellular functions of Mp myosin XI in tip growth, future research needs to visualize individual organelles, vesicles and Mp myosin XI in *Marchantia* rhizoids. Furthermore, a detailed comparative analysis of the functions of myosin XI in Arabidopsis and *Marchantia* might reveal how subfunctionalization and/or neofunctionalization of myosin XI occurred during land plant evolution.

## Experimental Procedures

### Plasmid construction

Total RNA was extracted from the thalli of Takaragaike‐1, a wild‐type accession of *M*.* polymorpha* (Ishizaki *et al*., 2008), and cDNA was prepared as described in Kanazawa *et al*. (2016). Coding sequences of Mp myosin XI were amplified by PCR from the cDNA using the following primer sets: 5′‐CACCATGGCGACTGCAAATATTTCAATCGG‐3′ and 5′‐TCATCTCAAATTAGCCACACGAAGCTCG‐3′ for Mp8g04010.1 and 5′‐CACCATGGCGACTGCAAATATTTCAATCGGATCGC‐3′ and 5′‐TTACTCATGCTGTGGTTG‐3′ for Mp8g04010.2. The tail domain of Mp8g04010.2 was amplified by PCR from the pENTR^TM^/Mp8g04010.2 with the following primer sets: 5′‐CACCATGAAGCTCAAAATGGCTGCAAAGG‐3′ and 5′‐TTACTCATGCTGTGGTTGTA‐3′. The amplified fragments were then subcloned into pENTR^TM^/D‐TOPO (Thermo Fisher Scientific, Waltham, MA, USA, www.thermofisher.com), according to the manufacturer's instructions.

To examine the molecular properties of Mp myosin XI using *in vitro* mechanical and enzymatic analyses, baculovirus transfer vectors for Mp myosin XI‐Full and Mp myosin XI‐MD constructs were generated using site‐directed mutagenesis PCR, as follows: Mp myosin XI‐Full and Mp myosin XI‐MD fragments were amplified by PCR from the cDNA of Mp8g04010.2 with the primer sets 5′‐AAACCATGGCGACTGCAAATATTT‐3′ and 5′‐TTTACCGGTCTCATGCTGTGGTTGTAAAAA‐3′ as well as 5′‐AAACCATGGCGACTGCAAATATTTC‐3′ and 5′‐TTTACCGGTTGCCGCATTGCTTAGAAG‐3′, respectively. The PCR products were created with a *Nco*I site at the 5′‐end and an *Age*I site at the 3′‐end of nucleotide 4608 (Mp myosin XI‐Full) or 2214 (Mp myosin XI‐MD). They were then ligated to the *Nco*I–*Age*I fragment of an N‐terminal‐modified pFastBac MD‐Vneck (Ito *et al*., 2007). The resulting proteins (Mp myosin XI‐Full and Mp myosin XI‐MD) had an N‐terminal tag (MDYKDDDDKRSMNSRPA) containing the FLAG tag (DYKDDDDK), amino acid residues 1–1536 (Mp myosin XI‐Full) or 1–738 (Mp myosin XI‐MD), a C‐terminal tag (GGGEQKLISEEDLHHHHHHHH) containing a flexible linker (GGG), a Myc‐epitope sequence (EQKLISEEDL) and a (His)8 tag. They were expressed in insect cells (High Five^TM^, Thermo Fisher Scientific) and affinity‐purified as previously described by Ito *et al*. (2007, 2009).

Mp myosin XI‐Full or Mp myosin XI‐tail in the pENTR/D‐TOPO cloning vector was transferred into pGWB406 or pGWB461 (Nakagawa *et al*., 2007) by site‐specific recombination with Gateway LR Clonase (Invitrogen Corporation). The myosin XI‐2, XI‐J and XI‐K tail domains were amplified and subcloned into pENTR^TM^/D‐TOPO as described in Haraguchi *et al*. (2018). These At myosin XI‐tails in the pENTR/D‐TOPO cloning vector were transferred into pGWB406 for transient expression in Arabidopsis suspension culture cells (Alex) (Mathur and Koncz, 1998).

A fluorescent marker for F‐actin visualization, Lifeact‐TagRFP (Era *et al*., 2009), an ER‐targeting marker, SP‐TagRFP‐HDEL (Mitsuhashi *et al*., 2000), a Golgi body‐targeting marker, ST‐mRFP (Boevink *et al*., 1998) and the peroxisome‐targeting marker TagRFP‐H1 (Mano *et al*., 1999) were individually subcloned into pENTR^TM^/D‐TOPO. These fluorescence markers in the donor vector were used for cloning into the binary vectors pGWB402 or pGWB502, containing the CaMV 35S promoter for the transient expression in Arabidopsis suspension culture cell (Alex).

The 3 kb sequences upstream of the At myosin XI‐2 and XI‐K open reading frame start codons with their putative promoters were amplified, as previously described in Haraguchi *et al*. (2018). XI‐Kpro‐GFP‐Mp myosin XI and XI‐2pro‐GFP‐Mp myosin XI were constructed using triple‐template PCR and subcloned into pENTR^TM^/D‐TOPO, respectively. The donor vector with XI‐Kpro‐GFP‐Mp myosin XI or XI‐2pro‐GFP‐Mp myosin XI was used for cloning into pFAST‐G01 (Inplanta Innovations, Tokyo, Japan, https://www.inplanta.jp/en.html) via site‐specific recombination using the Gateway LR Clonase II enzyme mix.

### ATPase activity and *in vitro* actin‐gliding assays

Steady‐state ATPase activity and *in vitro* actin‐gliding assays were measured and performed at 25°C, as previously described in Ito *et al*. (2007) and Haraguchi *et al*. (2014, 2016).

### Plant materials and growth conditions

*Arabidopsis thaliana* ecotype Columbia‐0 was used as the wild‐type plant. The quadruple knockout line (4KO) for myosin XI (*xi‐1 xi‐2 xi‐k xi‐i*) was described in a previous study (Peremyslov *et al*., 2010). The seeds were sown on rockwool (Yamato Plastic Co., Nara, Japan, http://www.yamato-plastic.co.jp/), irrigated with 1/2000 Hyponex solution and chilled for 48 h at 4°C in the dark. Plants were grown at 23°C with 80% relative humidity under 60 μmol m^–2^ sec^–1^ photon flux light conditions using a 16 h day/8 h night cycle. The *M*.* polymorpha* wild‐type accession Takaragaike‐1 was grown on 1/2 Gamborg's B5 medium containing 1% agar and 1% sucrose at 22°C under continuous white light (approximately 50 μmol m^–2^ sec^–1^ photon flux).

### Transient expression of Arabidopsis suspension culture cells (Alex)

The transient expression in the Arabidopsis suspension culture cell line Alex was aseptically performed using the following procedure. The constructs were transformed into *Agrobacterium tumefaciens* strain GV3101:pMP90 by electroporation using a Gene Pulser (Bio‐Rad Laboratories, Inc., Hercules, CA, USA, https://www.bio-rad.com/). Transformed *Agrobacterium* was cultured in LB medium containing 100 μg ml^−1^ spectinomycin and 100 μg ml^−1^ rifampicin until the OD_600_ reached 0.5. The bacteria were suspended in modified Murashige and Skoog medium and inoculated into 2‐day‐old Arabidopsis Alex cells using 2 mg ml^−1^ acetosyringone for efficient infection. Cells were incubated with gentle agitation at 23°C in dark conditions. To inactivate the bacteria, we added 20 mg ml^−1^ claforan (nacalai tesque, Kyoto, Japan, https://www.nacalai.co.jp/global/) into the 3‐day‐old Arabidopsis Alex cells. Microscopy observations were performed 3 or 4 days after *Agrobacterium* inoculation (Saito *et al*., 2011).

### Phalloidin staining for F‐actin

Petioles from the first leaf were harvested from 21‐day‐old Arabidopsis plants and cut into small pieces using dissecting scissors. Petioles were vacuum‐fixed for the first 5 min and incubated with 2% (w/v) paraformaldehyde in a fixation buffer (50 mm PIPES, pH 7; 10 mm EGTA; 5 mm MgSO_4_, pH 7.0; 0.05% Triton X‐100) for 2 h. Samples were then stained using 200 nm Alexa Fluor 488 phalloidin (Thermo Fisher Scientific) in a fixation buffer containing 0.03% Triton X‐100 for 1 h in dark conditions. The staining solution on the surface of samples was then removed by washing several times with the fixation buffer. Epidermal cells of the petiole were observed under a confocal laser scanning microscope. The resulting images were then processed using Image J software (NIH). The processed images were measured using two parameters: sinθ for orientation and NormAvgRad for parallelness using the LPixel ImageJ Plugins (LPixel Inc., Tokyo, Japan, https://lpixel.net/en/).

### MitoTracker Orange staining

Three‐day‐old *Marchantia* rhizoids were stained with 50 nm MitoTracker Orange CMTMRos (Invitrogen) in 1/2 Gamborg's B5 medium at 20–25°C for 30 min. The samples were then washed and mounted in a glass‐bottomed dish with 1/2 Gamborg's B5 medium for observation.

### Arabidopsis transformation

The XI‐Kpro‐GFP‐Mp myosin XI and XI‐2pro‐GFP‐Mp myosin XI in pFAST‐G01 were transformed into the *Agrobacterium* strain GV3101:pMP90 by electroporation using a Gene Pulser (Bio‐Rad). They were then introduced into 4KO (*xi‐1 xi‐2 xi‐k xi‐i*) plants using the floral dip method (Clough and Bent, 1998). First‐generation transformed Arabidopsis seeds (T_1_) were identified and selected with a fluorescence stereomicroscope (SZX10, Olympus, Tokyo, Japan, https://www.olympus-global.com/).

### Confocal laser scanning microscopy

The fluorescence images were inspected by spinning‐disk confocal microscopy (CSU‐X1 or CSU‐W1, Yokogawa, Tokyo, Japan, https://www.yokogawa.com/) equipped with a sCMOS camera (ORCA FLASH4.0 V2, Hamamatsu Photonics, Shizuoka, Japan, https://www.hamamatsu.com/) to obtain *z*‐stack or time‐lapse images. The GFP and Alexa Fluor 488 fluorescences were observed using excitation and emission wavelengths of 488 nm and 475–575 nm, respectively. GFP‐Mp myosin XI‐Full streaming was recorded at 500 ms intervals in Arabidopsis Alex cells and Arabidopsis plants. TagRFP and mRFP fluorescences were observed using excitation and emission wavelengths of 561 nm and 580–700 nm, respectively. The resulting images were processed using the Image J (NIH, https://imagej.nih.gov/ij/) or Metamorph software (Molecular Devices, San Jose, CA, https://www.moleculardevices.com/). Cytoplasmic streaming was captured using differential interference contrast (DIC) images in 500 ms intervals for 30 sec for 15 cells per genotype. The maximum velocity of cytoplasmic streaming was determined by tracing the movements of clearly visible particles moving smoothly for distances of >2.5 μm using the Image J software. MitoTracker Orange was observed under a confocal microscope (LSM780, Carl Zeiss, Oberkochen, Germany, https://www.zeiss.com/). Samples were excited by a 561 nm laser and emitted fluorescence between 566 nm and 627 nm. For the time‐series observations of DIC and MitoTracker Orange in *Marchantia* rhizoids, images were collected every 10 sec for 10 min. The maximum velocity of mitochondria was determined by tracing the continuous movements of MitoTracker Orange particles using Image J software for 40 particles from 12 rhizoids.

## Statistical Analyses

Students' *t‐*test was used to assess differences between two samples.

## Accession Numbers

The protein sequences of Mp8g04010.1 have been submitted to GenBank under accession number PTQ46267.1. The mRNA and protein sequences of Mp8g04010.2 have been submitted in GenBank under accession numbers AB516314.1 and PTQ46266.1.

## Author Contributions

Author contributions were as follows: MT, TU, KI and ZD designed the experiments; ZD, MT, TK, AE, TH and TK performed the experiments; ZD, MT, TK, TU, KI and TH wrote the article.

## Conflict of Interest Statement

The authors have no conflicts of interest to declare.

## Supporting information


**Video S1.** Motility of GFP‐Mp myosin XI‐Full in an Arabidopsis cultured cell.

**Video S2.** Cytoplasmic streaming in petiole epidermal cells of wild‐type plants.

**Video S3.** Cytoplasmic streaming in petiole epidermal cells of quadruple At myosin XI knockout mutant plants.

**Video S4.** Cytoplasmic streaming in petiole epidermal cells of 4KOR‐XI‐Kpro plants.

**Video S5.** Cytoplasmic streaming in petiole epidermal cells of 4KOR‐XI‐2pro plants.

**Video S6.** Motility of GFP‐Mp myosin XI‐Full in petiole epidermal cells of 4KOR‐XI‐Kpro plants.

**Video S7.** Motility of GFP‐Mp myosin XI‐Full in petiole epidermal cells of 4KOR‐XI‐2pro plants.

**Video S8.** Motility of GFP‐Mp myosin XI‐Full in petiole epidermal cells of 4KOR‐XI‐2pro plants. Images were taken every 0.5 s.

**Video S9.** Cytoplasmic streaming in *Marchantia* rhizoids. Images were taken every 10 sec.

**Video S10.** Motility of mitochondria in *Marchantia* rhizoids. Images were taken every 10 sec.

**Figure S1.** Schematic diagram of general morphology of *Marchantia polymorpha* myosin XI used in the present study.
**Figure S2.** Pharmacological treatment.
**Figure S3.** Co‐localization and movement of *Marchantia polymorpha* myosin XI and endoplasmic reticulum in Arabidopsis cultured cells.
**Figure S4.** Confocal images of GFP‐fused *Marchantia polymorpha* myosin XI and fluorescent organelle markers in Arabidopsis cultured cells.

## Data Availability

All relevant data can be found within the manuscript and its supporting materials.
