# Supplementary material for: Characterization of ancestral myosin XI from Marchantia polymorpha by heterologous expression in Arabidopsis thaliana
Source: Plant J. 2020 Aug 6;104(2):460–73. doi: 10.1111/tpj.14937 (PMC7689712; doi:10.1111/tpj.14937)
Supplement: Supplementary file 11 — Figure S1. Schematic diagram of general morphology of Marchantia polymorpha myosin XI used in the present study. Figure S2. Pharmacological treatment. Figure S3. Co‐localization and movement of Marchantia polymorpha myosin XI and endoplasmic reticulum in Arabidopsis cultured cells. Figure S4. Confocal images of GFP‐fused Marchantia polymorpha myosin XI and fluorescent organelle markers in Arabidopsis cultured cells. [file TPJ-104-460-s011.docx]

**Supporting Information**


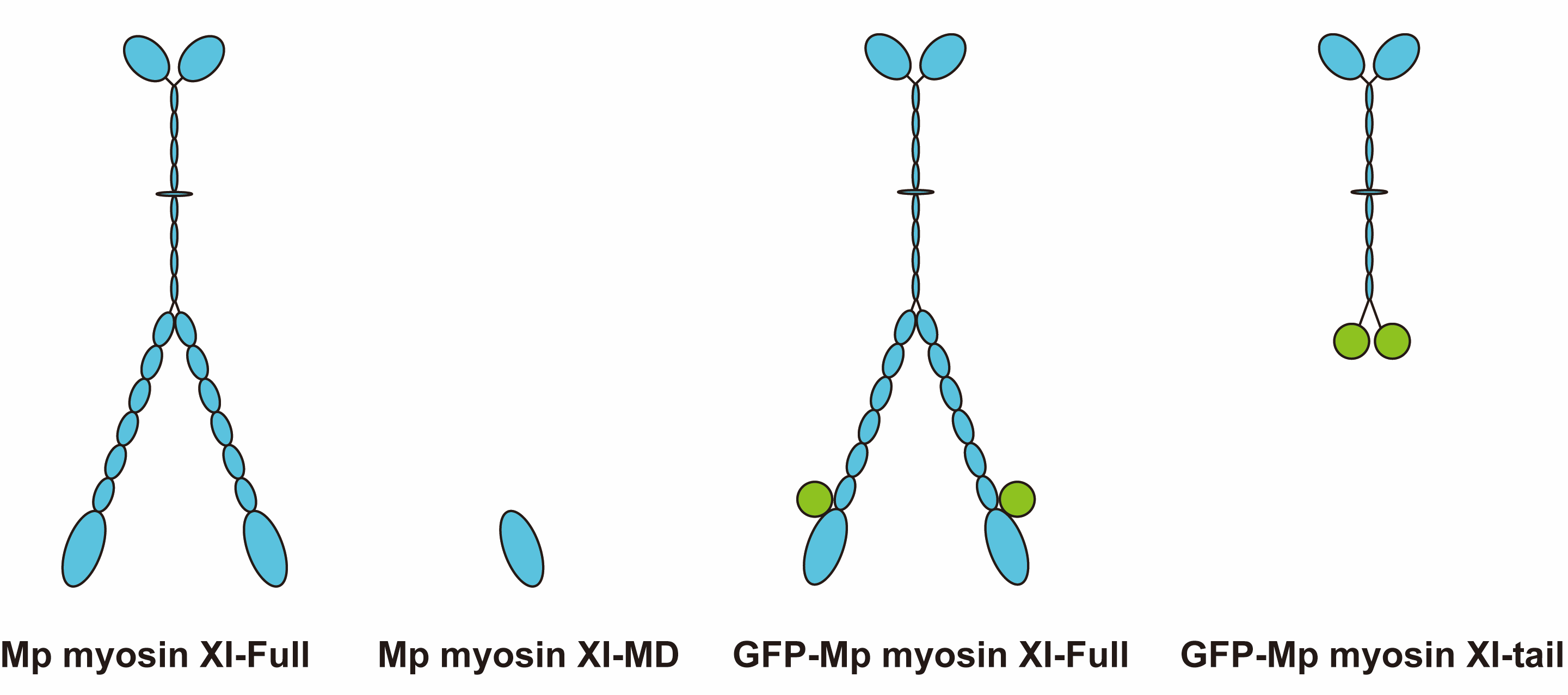


**Figure S1. Schematic diagram of general morphology of Mp myosin XI used in the present study.**

Diagrams showing domain structure of Mp myosin XI-Full, motor domain of Mp myosin XI (Mp myosin XI-MD), GFP-fused full-length Mp myosin XI (GFP-Mp myosin XI-Full) and GFP-fused Mp myosin XI-tail domain (GFP-Mp myosin XI-tail). sGFP (green sphere) was fused to the N terminus of Mp myosin XI-Full and Mp myosin XI-tail.


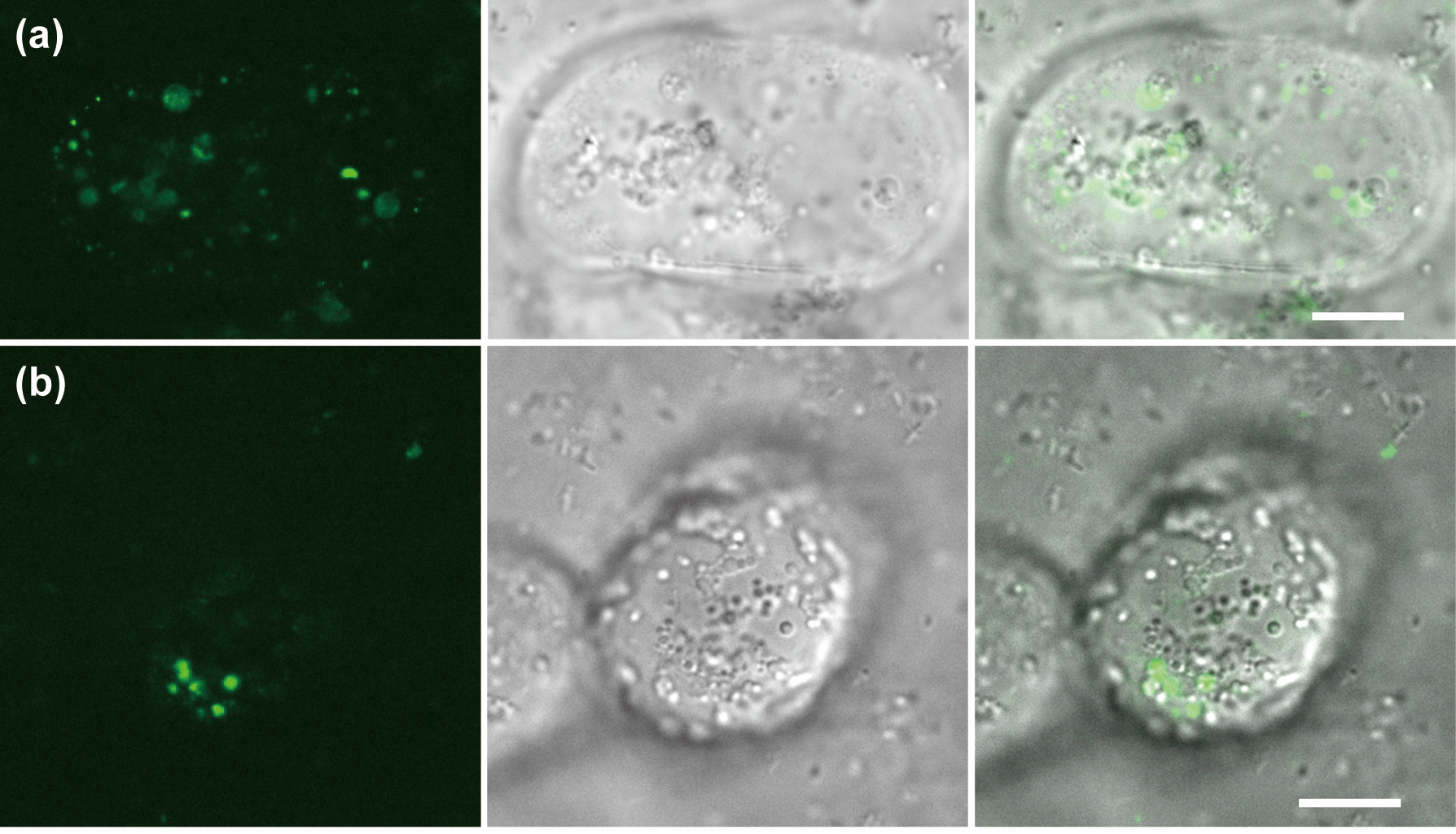


**Figure S2 Confocal images of GFP-fused Mp myosin XI with latrunculin B treatment in Arabidopsis cultured cell.**

Pharmacological treatment of Arabidopsis Alex cells expressing GFP-Mp myosin XI-Full with an inhibitor of actin polymerization, latrunculin B for 10 min (a) and 60 min (b).


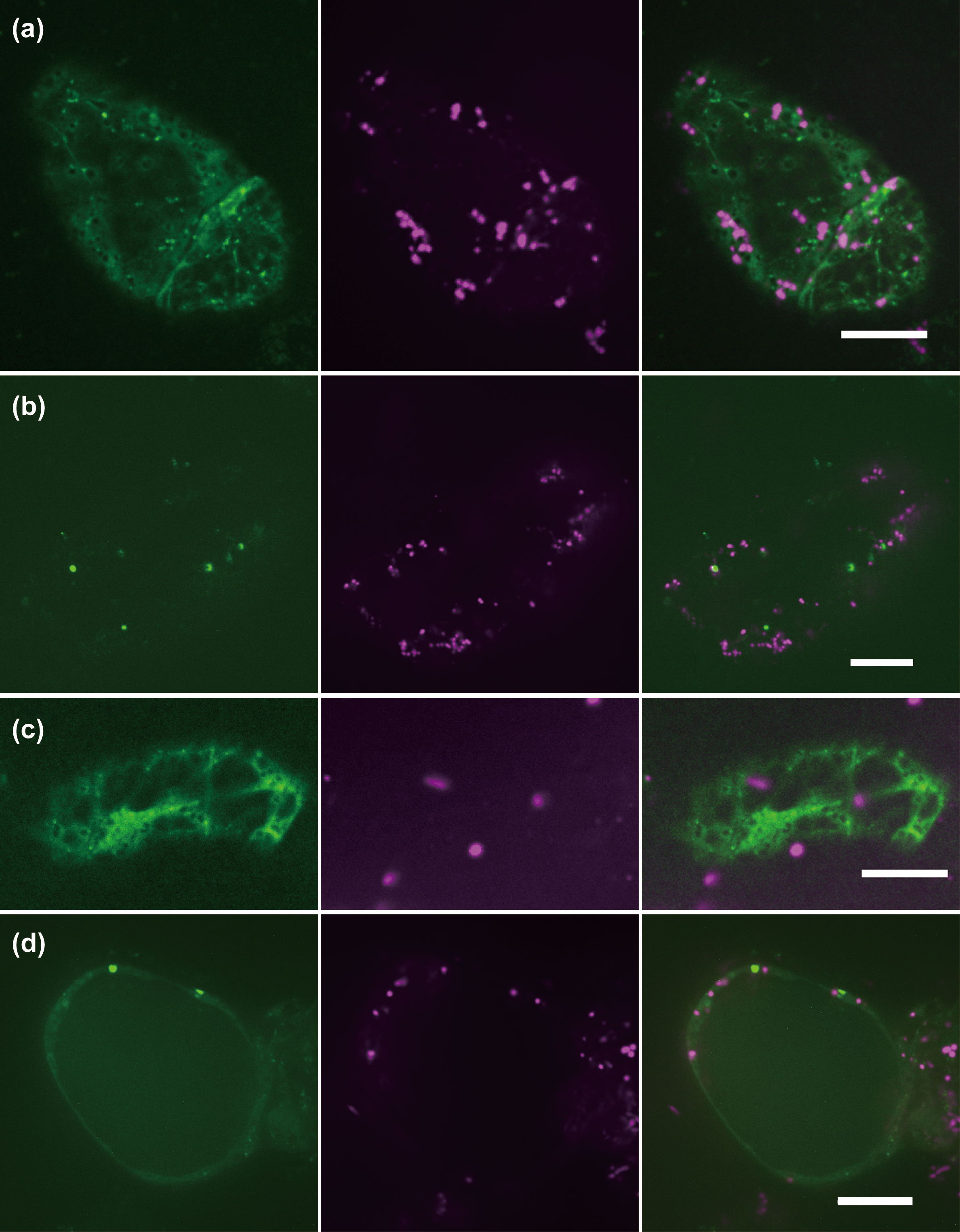


**Figure S3. Confocal images of GFP-fused Mp myosin XI and fluorescent organelle markers in Arabidopsis cultured cell.**

(a) Co-expression of GFP-Mp myosin XI-Full (green) and ST-mRFP, a Golgi body marker (magenta). (b) Co-expression of GFP-Mp myosin XI-tail (green) and ST-mRFP (magenta). (c) Co-expression of GFP-Mp myosin XI-Full (green) and TagRFP-H1, a peroxisome marker (magenta). (d) Co-expression of GFP-Mp myosin XI-tail (green) and TagRFP-H1, a peroxisome marker (magenta). Bars = 10 μm.

**Movies**

Movie S1. Motility of the GFP-Mp myosin XI-Full in the Arabidopsis cultured cell. Images were taken every 0.5 s. 1 × real time, bar = 10 μm.

Movie S2. Co-localization and movement of Mp myosin XI-binding structures and ER strands in Arabidopsis cultured cell. Images of GFP-Mp myosin XI-Full (green) and TagRFP ER marker (magenta) were taken every 0.5 s. 1 × real time, bar = 10 μm.

Movie S3. Cytoplasmic streaming in the petiole epidermal cell of wild-type plants. Images were taken every 0.5 s. 1 × real time, bar = 10 μm.

Movie S4. Cytoplasmic streaming in the petiole epidermal cell of 4KO plants. Images were taken every 0.5 s. 1 × real time, bar = 10 μm.

Movie S5. Cytoplasmic streaming in the petiole epidermal cell of 4KOR-XI-Kpro plants. Images were taken every 0.5 s. 1 × real time, bar = 10 μm.

Movie S6. Cytoplasmic streaming in the petiole epidermal cell of 4KOR-XI-2pro plants. Images were taken every 0.5 s. 1 × real time, bar = 10 μm.

Movie S7. Motility of the GFP-Mp myosin XI-Full in the petiole epidermal cell of 4KOR-XI-Kpro plants. Images were taken every 0.5 s. 1 × real time, bar = 10 μm.

Movie S8. Motility of the GFP-Mp myosin XI-Full in the petiole epidermal cell of 4KOR-XI-2pro plants. Images were taken every 0.5 s. 1 × real time, bar = 10 μm.

Movie S9. Cytoplasmic streaming in Marchantia rhizoids. Images were taken every 10 sec. 50 × real time, bar = 10 μm.

Movie S10. Motility of mitochondria in Marchantia rhizoids. Images were taken every 10 sec. 50 × real time, bar = 10 μm.
